# Controlled Drug Delivery Device for Cornea Treatment and Novel Method for Its Testing

**DOI:** 10.3390/ph16040505

**Published:** 2023-03-28

**Authors:** Pavel Urbánek, Pavol Šuly, Jakub Ševčík, Barbora Hanulíková, Ivo Kuřitka, Tomáš Šopík, Mehrdad Rafat, Pavel Stodůlka

**Affiliations:** 1 Centre of Polymer Systems, Tomas Bata University in Zlín, Trida Tomase Bati 5678, 76001 Zlin, Czech Republic; 2 LinkoCare Life Sciences AB, 16975 Stockholm, Sweden; 3 NaturaLens AB, 58330 Linköping, Sweden; 4 Gemini Eye Clinic, U Gemini 360, 76001 Zlin, Czech Republic

**Keywords:** controlled drug release, drug delivery device, cornea treatment

## Abstract

A new solution for local anesthetic and antibiotic delivery after eye surgery is presented. A contact lens-shaped collagen drug carrier was created and loaded by Levofloxacin and Tetracaine with a riboflavin crosslinked surface layer, thus impeding diffusion. The crosslinking was confirmed by Raman spectroscopy, whereas the drug release was investigated using UV-Vis spectrometry. Due to the surface barrier, the drug gradually releases into the corneal tissue. To test the function of the carrier, a 3D printed device and a new test method for a controlled drug release, which mimics the geometry and physiological lacrimation rate of the human eye, were developed. The experimental setup with simple geometry revealed that the prepared drug delivery device can provide the prolonged release profile of the pseudo-first-order for up to 72 h. The efficiency of the drug delivery was further demonstrated using a dead porcine cornea as a drug recipient, without the need to use live animals for testing. Our drug delivery system significantly surpasses the efficiency of antibiotic and anesthetic eyedrops that would have to be applied approximately 30 times per hour to achieve the same dose as that delivered continuously by our device.

## 1. Introduction

Modern ophthalmology can solve many eye conditions with well-established procedures for the treatment of, e.g., cataracts or myopia, for improvement of vision and life without glasses or even corneal transplantation for the restoration of vision in a blind eye. In all cases, when a lens or implant is inserted or when the cornea is replaced with a donor graft [1] or artificial prosthesis [2,3], a certain invasive interaction with corneal tissue is unavoidable. Since the cornea, the eye-protecting barrier, is the most sensitive tissue in the human body, with the highest density of nerve fibers, any intervention (surgery, injury, disease) causes significant pain [4]. Effective treatment of the corneal tissue is, therefore, highly desired, involving not only analgesia but also inflammation prevention. The most used method—eye-drop treatment—requires frequent application because its efficacy is low due to several tissue barriers that must be overcome on the way from the anterior to the posterior segment of the eye via the corneal or conjunctival route [5]. In addition, blinking, lacrimation, and rapid draining of the drop from the eye surface by tears decrease the residence time of the drug very quickly [6]. Eyedrops thus provide a maximum bioavailability of the delivered drug of only 5%, as a result of the low thermodynamic activity of the permeating drug through eye barriers by passive diffusion [7,8]. Moreover, adherence to eyedrop treatment by patients and the administering of the drops itself has appeared to be very difficult, especially for elderly people [9]. To ensure the proper intensity of a prescribed treatment after eye surgery or long-term treatment, controlled topical drug delivery with a spontaneous release of an active substance is a satisfying solution.

Soft hydrogel materials (polyethylmethacrylate, poly-2-hydroxyethylmethacrylate, polymethacrylate) or silicone (polydimethylsiloxane) polymers have been used for the manufacture of contact and intraocular lenses for years [10]. Hydrogels form a network that can embed a drug. Therapeutic contact lenses (TCL) can, therefore, provide the proper concentration of a desired substance on the ocular surface. For instance, a contact lens prepared from a solution of glycolide:L-Lactide (85:15) with dexamethasone by spin coating in the mold has proved its efficiency for several days and provided a higher concentration of the corticosteroid in eye parts, including the posterior chamber (e.g., retina) in comparison with eyedrops when studied in vivo in rabbits [11]. Conjunctivitis in rabbits was successfully treated by moxifloxacin/hyaluronic acid (HA) in lenses prepared by polymerization of several monomers: hydroxyethyl methacrylate, methacrylic acid, 1-Hydroxycyclohexyl phenyl ketone, and ethylene glycol di-methacrylate. Moxifloxacin/HA was loaded in the lens’s peripheral rings; therefore, no effect on transparency in the middle of the lens was observed [12]. However, maintaining the critical properties of the contact lenses, such as optical transparency, mechanical properties, and oxygen and ion permeability—which is very important for the prevention of hypoxia and inflammation of the corneal tissue—is a challenge [13]. Several methods of preparation [14,15], such as (i) soaking, which provides only short periods of hours of drug release, (ii) soaking with Vitamin E as a diffusion barrier for a longer releasing profile, (iii) molecular imprinting, nanoparticle, and micelle systems for prolonged delivery lasting weeks, have been tested to treat glaucoma [16], dry eyes [17], or eye conditions connected with diabetes [18]. Other systems that do not need to fulfill all conditions required for contact lenses, e.g., transparency, have already been introduced. The system of polyethylene glycol (PEG) hydrogel with dexamethasone particles has been prepared to form Dextenza intracanalicular insert (Ocular Therapeutix) for topical treatment of the eye [19]. Gradual hydrolysis of the PEG hydrogel enables a slow release of entrapped corticosteroid for up to one month [20]. Hydrophobic liquid suspension is used for the treatment of a posterior chamber of the eye as it can be injected where needed and where eyedrops have no effect. The suspension of acetyl triethyl citrate can accommodate small droplets with molecules of drugs, e.g., corticosteroids, as in the case of DEXYCU^®^, YUTIQ^®^, ILUVIEN^®^, RETISERT^®^ by EyePoint Pharmaceuticals. When injected they form a biodegradable spherule of 2 mm via hydrophobic interactions, and the active substance is then released gradually for several months [21]. The research in this field is very active and even though some systems have already been approved by FDA (as in the examples above), other solutions for topical treatment are still under investigation. Solid lipid nanoparticles of stearic acid with Levofloxacin have been reported for the treatment of conjunctivitis. The nanoparticles with a several-hour-long two-phase release profile (initial burst release and sustainable release) showed in vitro antibacterial activity [22]. Interesting results have been also achieved with silicon nanoparticles and lipids as hybrid carriers for small interfering RNA in the form of eyedrops with an intended use in the field of ocular gene therapy. In vivo study in mice has revealed that siRNA reached to corneal cells successfully, and it reduced the targeted protein expressions in corneal epithelium [23]. Controlled release approaches based on barriers from metal-organic frameworks [24] or based on liposomes [25] have also emerged. 

Further, the bioengineered collagen of animal origin has been at the center of attention because it is suitable for use as a drug carrier, and it also has great potential to become an artificial cornea. The collagen is thoroughly purified; therefore, all foreign cells and antigens are removed, and the material is safe for the patient for long-term use with a significantly reduced risk of a severe immune reaction [26]. The first positive results of clinical studies on a replacement of the cornea with the recombinant human collagen crosslinked with 1-ethyl-3-(3-dimethylaminopropyl)carbodiimide (EDC) were reported in 2010 [27]. Collagen devices have shown their potential when colonized by the recipient’s epithelial cells, keratocytes, and nerves [28,29] and some of them, e.g., Xenia^®^ by Gebauer Medizintechnik GmbH and LinkCor^®^ by LinkoCare Life Sciences AB, are now being under prospective and clinical studies [30,31]. 

Collagen drug carriers must be transparent, biocompatible, and have sufficient mechanical properties if they are supposed to be used as contact lenses or cornea implants. Crosslinking of the collagen fibers has proved to be a suitable way to improve toughness, even when outpatient surgery is conducted directly in the patient’s eye with the use of riboflavin [32,33]. This photosensitizer exposed to UV-A induces physical crosslinking via singlet oxygen formation and a reaction with the carbonyl group, which creates imidazolone intermediate that further reacts with the hydroxyl group (nucleophile) and forms crosslinks [34]. Free carbonyl groups in tissue, in contrast to amine groups, are essential for UV-A crosslinking [35]. The procedure is described within the Dresden protocol established by Wollensak et al. [36] that specifies the suitable concentration of riboflavin phosphate (0.1 wt%) in 20 wt% dextran solution, UV-A wavelength 370 nm, irradiance 3 mW/cm^2^ and time of the treatment 30 min. An almost two-fold and four-fold increase in Young’s modulus was achieved in porcine and human corneas, respectively [37]. Another source has reported an increase of Young’s modulus of the porcine cornea by 127% on average [38]. Another approach, chemical crosslinking of collagen, is a compromise between good mechanical properties provided by glutaraldehyde that is cytotoxic [39] and poorer mechanical properties, low crosslinking density, and poor resistance to enzymatic degradation attained by biocompatible crosslinkers, e.g., EDC and N-hydroxysuccinimide (NHS) [40]. As a crosslinker, l,4-Butanediol diglycidyl ether has worked for mechanical properties, but it decreased the biocompatibility of prepared hydrogel in comparison with the EDC/NHS system [41]. On the other hand, the porous character of porcine collagen crosslinked by EDC/NHS can retain substances and release them gradually, as has been shown with recombinant rat nerve growth factor beta [42] and vancomycin, while maintaining satisfactory biomechanical and optical properties important for implant [43]. The downsides of chemical crosslinking can be avoided by a preparation of collagen systems via a crosslinker-free gelation of pyrene conjugated dipeptide amphiphile of lysine and cysteine that creates a network and locks the collagen fibers in without any change in their functional groups but with an improvement in mechanical properties. Moreover, such dipeptide-collagen network is suitable for further functionalization of the implant for drug delivery [44].

In our research, a contact lens-shaped collagen corneal device with a crosslinked surface layer enabling a gradual drug release for an immediate post-surgical treatment was prepared and studied ex vivo using a home-build device imitating a formation of tears on a dead porcine eye and enabling the monitoring of the drug release with spectroscopic methods. The results on the soaking capacity of the collagen carrier, the kinetics of the releasing process, and the amount of released substance—an anesthetic, Tetracaine and an antibiotic, Levofloxacin—were obtained. In addition, we developed a 3D printed model simulating the eye bulb geometry and lacrimation, which allows tracking the drug release from the carrier. Moreover, the delivery of the drug into the cornea excised from a dead animal eye can be examined using the model. The suitability of use of a dead porcine eye for this experiment was assumed from the literature review, as various model experiments with dead porcine eyes, such as a description of light propagation in the eye for understanding the eye parameters in laser treatment [45], a description of changes on a surface and cornea of a dry eye [46], a study of the widespread use of a crosslinking procedure of cornea [47], experiments on novel ways to distribute drugs with large molecules by microneedle-lens in the cornea [48], without any risk for patients, have been conducted. Similar models of dry eye with lacrimation and blinking system have been found beneficial as the experimental conditions can be fully controlled, no live animal models are needed, and the results are reproducible [49,50]. Nevertheless, these earlier models use whole enucleated dead animal eyes, which limits their practical use.

## 2. Results and Discussion

### 2.1. Preparation and Characterization of Drug Carrier

**Figure 1:**
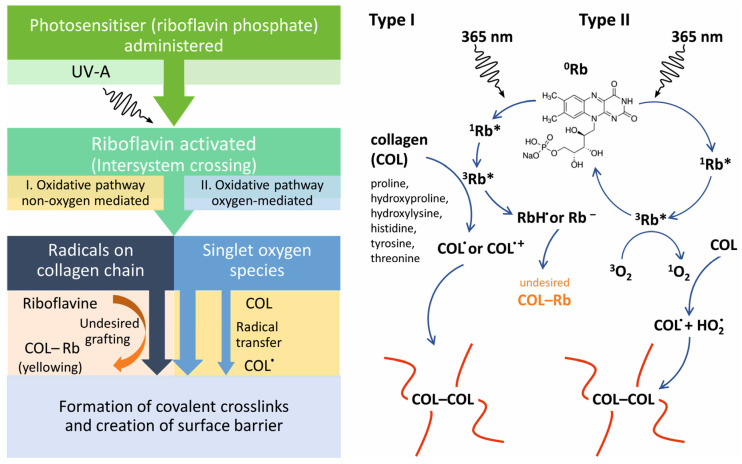
Scheme of crosslinking barrier creation on the surface of the lens-shaped collagen drug carriers (**left**), chemistry of collagen crosslinking (**right**). Key: COL—collagen; Rb—riboflavin; Rb*—activated riboflavin.

The collagen drug carrier was prepared by creating a crosslinked barrier on the collagen carrier surface. The procedure described in the Dresden protocol [51] was used with partially modified parameters as indicated in the Experimental part of this study. Possible mechanisms of collagen crosslinking are schematized in Figure 1. Among other substances, collagen contains proline, hydroxyproline, hydroxylysine, histidine, tyrosine and threonine, which are amino acids with chemical structures prone to radical reactions within Type I or Type II oxidative pathways. On the other hand, we do not consider glycosylative crosslinking within proteoglycans and collagen fibrils, as we work with medical-grade atelocollagen of high purity [33,34,52,53]. 

Absorption of UV-A by riboflavin triggers oxidative and oxygen-mediated pathways that lead to the formation of collagen crosslinks. An excited riboflavin phosphate molecule undergoes intersystem crossing into a long-living triplet state. In the non-oxygen mediated oxidative pathway (Type I), the energy is transferred from the riboflavin molecule in the triplet state to the collagen macromolecule via hydrogen or electron transfer, producing collagen radicals or radical cations that enable crosslinking. As a result, covalent links between side groups of the collagen’s amino acid mer units are created. Both intramolecular and intermolecular linkages can be produced, the latter resulting in chemical cross-linking. The intramolecular linkages may cause physical crosslinking if the polymer chain loops are concatenated. Nevertheless, riboflavin is also altered, creating a riboflavin radical or radical anion. These species may be grafted onto the collagen chains, causing undesirable yellowing of the material. They also may react with other low molecular species, including the APIs, thus forming undesired byproducts. Therefore, a thoroughly performed rinsing step after crosslinking of the drug carrier and the HPLC control of eventual chemical alteration of used APIs are vital. In the second oxygen-mediated oxidative pathway (Type II), the energy from the riboflavin molecule in the triplet state is transferred to a triplet ground state oxygen molecule. This process generates singlet oxygen (^1^O_2_) and replenishes riboflavin. Singlet oxygen is a highly reactive species that reacts readily with the amino acids’ carbonyl groups and side groups in the collagen macromolecules, thus promoting collagen crosslinking. Hydroperoxyl radical is the byproduct of ^1^O_2_ reaction with the collagen substrate, another form of reactive oxygen species. Hydroperoxyl radicals may also be involved in further radical transfer and crosslinking of the polymer. Both ways trigger cascades of possible reactions, yielding chemical collagen crosslinking. In contrast to the Type I mechanism, the riboflavin molecule is regenerated in the ground state in each initial reaction cycle turnover and plays the role of a photocatalyst here. The oxygen-mediated pathway is much faster than the Type I reactions. Therefore, the Type II crosslinking mechanism dominates under the presence of oxygen. Nevertheless, if the oxygen concentration is depleted, the reactions belonging to Type II prevail [33,34,52,53].

**Figure 2:**
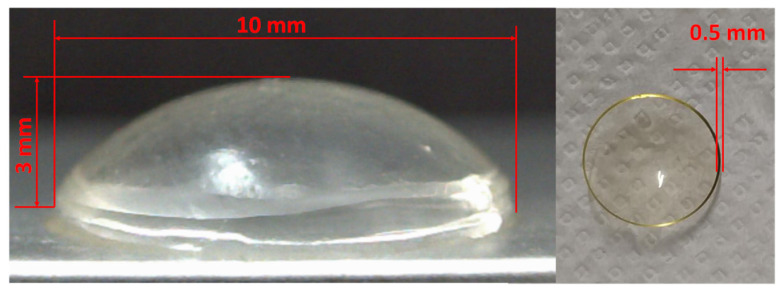
Photograph of the final collagen drug carrier with dimensions measured after drug saturation and formation of a crosslinked barrier on the surface.

The prepared collagen drug carrier of the contact lens shape soaked with API and crosslinked surface diffusion barrier is given in Figure 2. The shape of the drug carrier enables its use as a therapeutic contact lens with potential use as a corneal replacement. 

**Figure 3:**
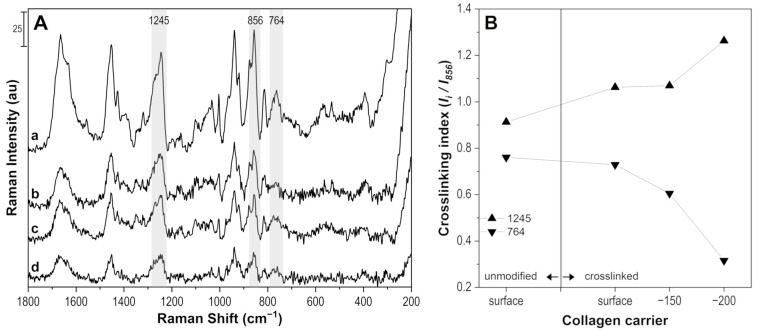
Raman spectra of swollen collagen carrier (a) unmodified, (b) crosslinked surface, (c) crosslinked 150 µm under the surface, and (d) 200 µm under the surface of the carrier (**A**—**left**). Crosslinking index obtained as I_i_/I_856_ of un/modified carrier at given depth from the surface, the lines are only guides to the eye (**B**—**right**).

The collagen carrier was characterized by the confocal Raman microscope to deliver proof of crosslinking of the surface layer by riboflavin in dextran solution under UV exposure. The carrier was analyzed in the swollen state (stored in DEMI water) and without any drug soaking, which enabled the characterization of only the effectivity of the crosslinking process. The spectra were collected before (unmodified) and after 15 min of a crosslinking process. Figure 3A represents the spectra of the swollen collagen carrier before (a) and after (b–d) crosslinking. Spectra b–d were measured as a depth profile, i.e., on the surface, 150 µm and 200 µm under the surface of the carrier, respectively. The spectral bands of unmodified collagen carrier (a) corresponds to vibrations of the amino acids that form its structure, i.e., mainly glycine, proline, alanine, hydroxyproline, and lysine. The bands are, therefore, assigned as follows [54]: 1663–1621 cm^−1^ Amide I; 1452 cm^−1^ ν_as_COO of Lys; 1424 cm^−1^ *δ*CH_3_; 1391 cm^−1^ ν_s_COO of lysine; 1321 cm^−1^ *δ*CH; 1245 cm^−1^ Amide III; 1161 cm^−1^ ωCH_2_, τNH_2_, δNNH_3_^+^ of hydroxylysine, 1104 cm^−1^ *δ*NCH of proline, 1032 cm^−1^ νCN of proline, 1001 cm^−1^ of phenylalanine; 940 cm^−1^ νCC skeleton of proline; 920 cm^−1^ C-COO-; 875 cm^−1^ νCC of glycine; 856 cm^−1^ νCC ring of proline; 814 cm^−1^ νCC skeletal; 764 cm^−1^ *δ*COO^−^; 535 cm^−1^ *δ*CCN, COO^−^ of alanine; 392 cm^−1^; and 304 cm^−1^ skeletal deformation. Due to the fact that the crosslinking mechanism is complex as it involves the creation of singlet oxygen and its reaction with the carbonyl group while forming imidazolone that further reacts with the hydroxyl group and forms crosslinks, there is no exclusive spectral band that would represent the newly created linkage. The changes in crosslinked collagen structure are usually observed as the changes in the intensity of spectral bands assigned to N-H and O-C=O groups [53]. In the case of the swollen carrier, the most pronounced effects were found for bands 1245 cm^−1^ (Amide III) and 764 cm^−1^ (δCOO^−^), as shown in Figure 3B with crosslinking index obtained as the ratio of the intensity of bands of interest (I_i_) and band at 856 cm^−1^ (I_856_) assigned to νCC of a proline ring. The changes connected with crosslinking were detected to a depth of 200 µm under the carrier surface with a total thickness of 500 µm.

**Figure 4:**
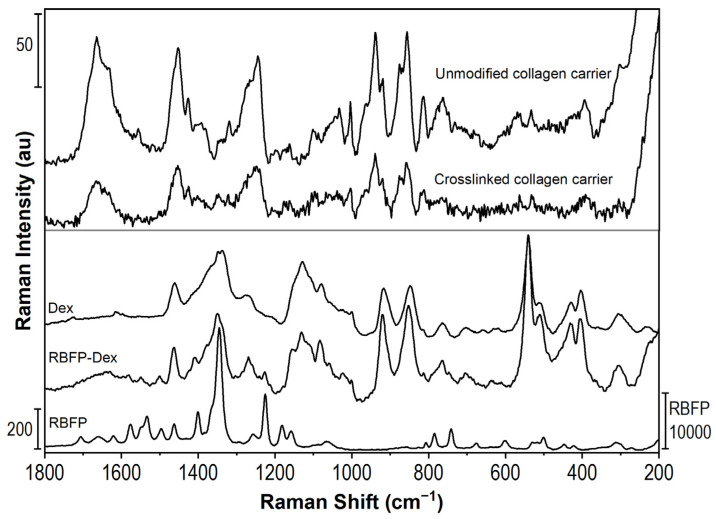
Raman spectra of swollen collagen carrier (unmodified and crosslinked) and dextran (Dex), riboflavin phosphate (RBFP) and the solution of RBFP in Dex.

Figure 4 compares the Raman spectra of the collagen carrier, riboflavin phosphate (RBFP), and dextran (Dex). The strongest band of riboflavin phosphate is 1346 cm^−1^ of in-plane rings deformation [55], whereas dextran is characterized by regions of about 1338 cm^−1^ of CH deformations, 1130 cm^−1^ of glycosidic bonds stretching (C-O-C, C-C-O, and C-C), and 917 cm^−1^ and 849 cm^−1^ of side groups (C-OH) deformations [56]. 

Raman spectra of the collagen carrier before and after crosslinking are qualitatively very similar, which confirms that the crosslinks are created between collagen amino acids. Therefore, the presence of riboflavin phosphate and dextran cannot be directly observed as a new band in the spectrum of the crosslinked carrier. This is understandable, as riboflavin is used in a low concentration of 0.1 wt% and serves as a photosensitizer that induces crosslinking reactions. Nevertheless, Raman spectroscopy results indicate that the surface layer of the collagen carrier was successfully modified by cross-linking.

### 2.2. Drug Release from the Collagen Carrier

**Figure 5:**
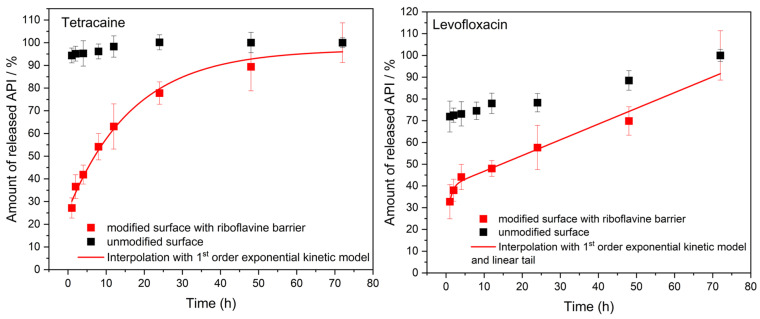
Tetracaine (**left**) and Levofloxacin (**right**) release profiles from unmodified (black) and surface-crosslinked (red) collagen drug carriers. The number of samples was 3 for each measurement.

The initial drug release characterization of prepared drug carriers was performed using our new 3D model. The collagen drug carriers were inserted into the apparatus without the porcine cornea to monitor the release for up to 72 h. The self-adherence of the drug carrier to the sample holder was such that no other fixtures were needed. The barrier effect of surface crosslinking modification of the drug carriers was examined. The release profiles plotted in Figure 5 clearly demonstrate the difference between modified and unmodified samples. The vast majority of loaded APIs is released from unmodified samples within the first hour of the test followed by a very slow release of the remaining portion of the drugs; the surface modification of the carrier has a pronounced effect on the APIs’ release profile. The release rate is significantly slowed down, and the profiles indicate a desirable gradual administration of the APIs to the simulated tear liquid. 

By modifying–crosslinking–the surface of the collagen carrier, the amount of Tetracaine released follows the mathematical model of first-order exponential growth. The data were interpolated using a model of the pseudo-first-order exponential release kinetics,

(1) $$y=y_{0}+A\cdot e^{\frac{x}{t}},$$

where the quantity *y* is the amount of released drug, *x* is time, *A* stands for the preexponential factor, and *t* is the characteristic time of the process. The mathematical fit agrees well with the experimental data (R^2^ = 0.98896). In the case of Levofloxacin, the release profile is different. It is clearly seen that the profile is composed of two processes. In the first part, the release follows the exponential growth formula of the pseudo-first-order release kinetics. However, the release continues to run linearly from the 12th hour to the end of the experiment, suggesting a constant rate release kinetics model. Therefore, the experimental data were fitted using a kinetic model which includes both the pseudo-first-order exponential and linear growth

(2) $$y=y_{0}+A\cdot e^{\frac{x}{t}}+k\cdot x,$$

where *y* is the amount of released drug and *x* is time. The parameter *A* stands for the preexponential factor, *k* is the slope of the linear part, and *t* is the characteristic time of the process. Also here, the mathematical model corresponds well to the obtained data (R^2^ = 0.97238). Parameters of both kinetic models are provided in Supplementary information (Figures S7 and S8). Approximately 45% of each API was released within the first 6 h of the experiment, indicating the initial drug distribution phase to the PBS. However, after this time, Tetracaine continued in an intensive release trend until the 24th hour of the experiment when 80% of this drug had been released. On the other hand, Levofloxacine was released more slowly from the collagen carrier, achieving approx. 70% of the released amount after 48 h. In both cases, the maximum time for drug release was 72 h when the entire amount of APIs was detected in the solution. Altogether, the results confirm the success of the developed crosslinking process in surface modification of the drug carriers. It is possible to achieve a suitable release rate of the APIs with respect to the application of the drug carrier under the conditions of the physiological lacrimation rate. 

**Figure 6:**
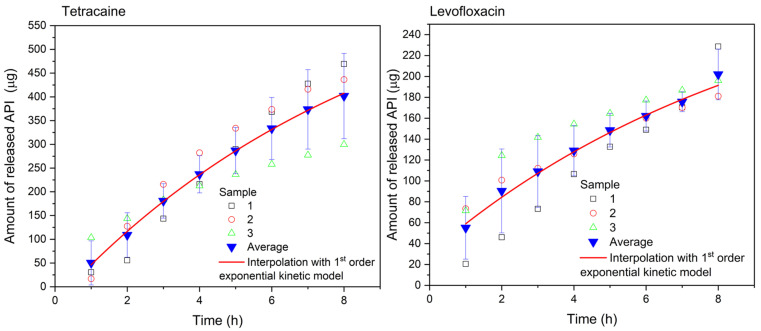
Tetracaine (**left**) and Levofloxacin (**right**) release profile. A loaded collagen drug carrier was placed on the top of the porcine cornea. The number of samples was 3 for each measurement.

To address the effectiveness of the APIs administration on the cornea tissue, the porcine cornea from a dead eye was used. The collagen drug carrier was placed on the top of the cornea mounted on the top of the sample holder spherical cap. The cornea specimen adhered to the substrate and the drug carrier adhered to the anterior corneal surface without any need of fixation. The PBS liquid simulating tears was delivered at the same physiological conditions as in the test of sole drug carriers. The liquid was collected and analyzed with UV-Vis spectrometry every hour. The Tetracaine and Levofloxacine release was monitored for only 8 h because of the loss of cornea freshness. The results are presented in Figure 6.

**Figure 7:**
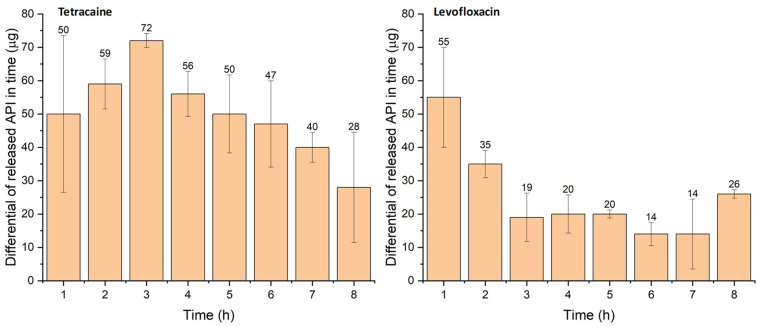
Dependence of released amount (µg) of drug from the carrier on time (**left**—Tetracaine, **right**—Levofloxacin). The number of samples was 3 for each measurement.

The release profiles obtained differ from the release profiles recorded for the sole drug carriers. Very flat curves corresponding to the pseudo-first-order release kinetics are observed, which testifies to the transport of a significant portion of the APIs to the cornea, whereas only a part of the APIs is released to the tear-simulating liquid. The mathematical interpolation of the pseudo-first-order model was done for both APIs according to Equation (1). The mathematical model agrees well with the experimental data (R^2^ = 0.99869 for Tetracaine, and R^2^ = 0.98804 for Levofloxacin). On the other hand, the exponential model with the Korsmeyer–Peppas (KP) component *b*·*t*^n^_r_ [57], where *b* represents the kinetic constant characteristic of the drug/polymer system, *t*_r_ is the release time, and n is the diffusional release exponent, fits the data obtained for Levofloxacin with greater accuracy (R^2^ = 0.99551)—see Figures S17 and S18. The Korsmeyer–Peppas model combined with the first-order exponential model seems to be an appropriate model for more structurally complicated substances and polymer carriers. Individual increments of the released drug per hour are shown in Figure 7. In the case of Tetracaine, the dose is released at a slightly increasing rate during the first three hours. After that, the release rate of Tetracaine begins to decrease, but tens of micrograms per hour of the drug are still released from the carrier over the whole period of the experiment. In contrast, the largest dose of Levofloxacin is released during the first hour, and then the increments are smaller but still in the order of tens of micrograms per hour.

To summarize, the collagen drug carrier releases continuously 30–70 µg of Tetracaine and 15–55 µg of Levofloxacin on average to the tear-simulating liquid every hour as confirmed for the 8 h of the experiment. The determined amount of released API, which is essential for the effect of treatment and bioavailability, is significantly higher when compared with API amount obtained with the eyedrops application. One drop (5 µL) of Tetracaine eyedrops with 2 wt% concentration of this API (usually 0.5–1 wt.%, but also 5 wt% drops are prepared) provides a maximum of 100 µg of Tetracaine. However, the absorption efficiency of the API from eyedrops must be considered because only 1%, i.e., 1 µg of Tetracaine can reach the eye tissue [57]. From such a comparison, the presented solution of collagen drug carrier with the modified surface is considered far more effective. 

**Figure 8:**
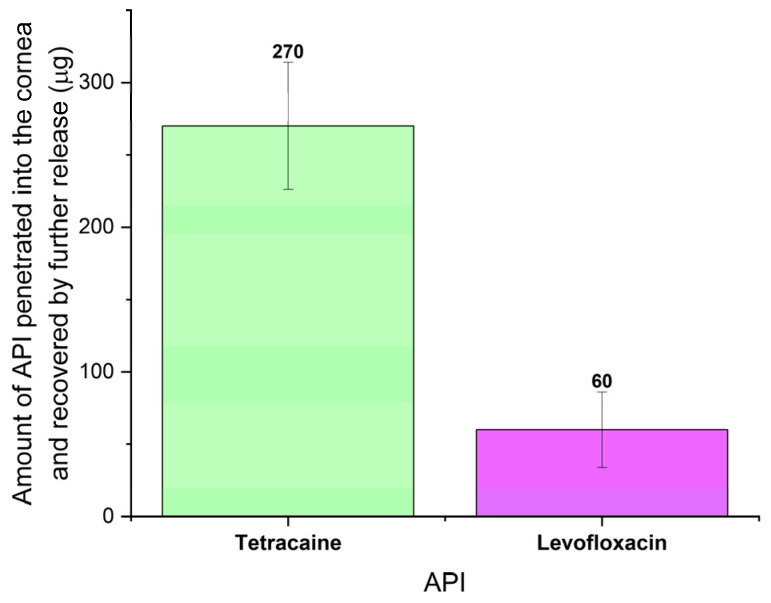
The amount of drug diffused into the cornea during an eight-hour experiment recovered in the subsequent leachate from the cornea. The number of samples was 3 for each measurement.

After the end of the drug release experiment, an analysis of the drug amount received by the cornea was performed. The cornea was inserted into a given volume of PBS, and UV-Vis spectra of the corneal leachate were measured after 24 h when the majority of the APIs was released and longer leaching did not bring any significant improvement. Based on the measured absorption and knowledge of the calibration curve, it was then possible to determine the amount of drug that passed into the cornea. As can be seen in Figure 8, 270 µg and 60 µg on average of Tetracaine and Levofloxacin, respectively, were leached from the cornea. These amounts represent 2/3 and 1/3 of the amounts of Tetracaine and Levofloxacin, respectively, released from the corresponding drug carrier to the tear-simulating liquid. The prepared collagen carrier can provide these amounts of studied API within 8 h of being in contact with the anterior surface of the cornea. This testifies to the enormous effectiveness and efficiency of the APIs administration to the corneal tissue. For example, when the amount of API obtained from UV-Vis spectra is compared with Tetracaine eyedrops (described in the previous paragraph), the same dose of Tetracaine would be reached only when applied 30 times per hour, representing one drop every two minutes. On the other hand, the toxic effect of Tetracaine, even after a topical application, requires caution in its use. The toxicity of Tetracaine is up to 10 times higher than that of Procaine [58].

### 2.3. Verification of Riboflavin Removal from the Crosslinked Barrier

**Figure 9:**
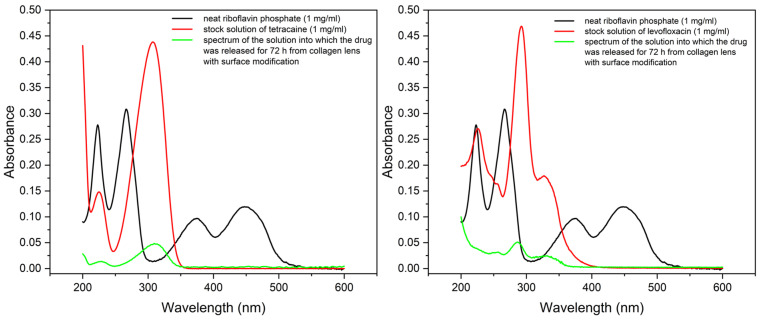
Comparison of UV-Vis spectra of neat riboflavin phosphate (black), neat Tetracaine (**right**, red), neat (**left**, red) and solutions after drug release from the crosslinked collagen carriers (green).

UV-Vis spectroscopy was also used to confirm the quality of the prepared crosslinked barrier on the surface of the collagen carriers. Riboflavin phosphate or riboflavin should not remain in the collagen carrier after crosslinking to avoid any further structural changes when exposed to UV radiation. Moreover, riboflavin would interfere with the optical spectra of the APIs in question. Figure 9 shows the absorption spectra of neat solutions of riboflavin phosphate, Tetracaine (on the left), Levofloxacin (on the right), and solutions with drugs released from the crosslinked collagen carrier after the 72 h-long experiment as described above in the drug release experiment section. 

As can be deduced from the spectra, riboflavin phosphate characterized by absorption maxima at 220, 260, 370, and 450 nm was not detected in solutions after either drug release experiment. Therefore, it can be stated that the prepared barrier on the surface of the collagen carrier is crosslinked with firm linkages, and the residual riboflavin was rinsed completely after the crosslinking process step. The drug carrier does not release any undesired chemical substances after crosslinking. 

Moreover, the particular API was confirmed in the solution after the drug release experiment with no significant change in the position of the absorption maxima (Tetracaine 225 and 310 nm, Levofloxacin 225, 290, 330 nm), indicating that the structure of the released drug was not altered, which was confirmed with HPLC/Q-TOF analysis.

### 2.4. Chemical Stability of API

The object of the drug stability study was to determine whether the drugs used (Tetracaine and Levofloxacin) changed their chemical structure after the entire process of collagen carrier impregnation (loading) and the subsequent release, or whether any degradation products were created. This is because the components in pharmaceutical forms could inappropriately interact if exposed to unusual conditions (e.g., UV radiation or induced chemical reactions), and further degradation of the API could occur, leading to its ineffectiveness. The study and verification of the chemical stability of the drugs were therefore carried out to demonstrate that Tetracaine and Levofloxacin are still the same drugs when released from the collagen carrier. Once the chemical structure of API after a drug release experiment is confirmed, the carrier can be considered a medical device and further clinical trials to verify the usability of the drugs themselves can be avoided. 

An HPLC/Q-TOF method has been developed for the determination of Tetracaine and Levofloxacin [59,60]. The pure drugs Tetracaine and Levofloxacin, used as reference samples, were measured in MS mode, while samples of solutions after drug release experiments were used to confirm particular drug product ions with MSMS mode. Chromatograms and MS spectra are provided in the Supplementary Information in Figures S9–S16. Both MSMS spectra of the product ions were identified with their reference MS spectrum, i.e., Tetracaine and Levofloxacin were confirmed in the solutions after drug release experiments with no change in their chemical structure. Moreover, no degradation products of either Tetracaine or Levofloxacin were identified in the samples of solution after the drug release experiment.

Therefore, the chosen advantageous procedure of using a drug approved by the national authority (SÚKL) appears to be viable, as no alterations in the chemical structure of the drugs during the entire process of the collagen drug carrier preparation were found. In other words, the released drugs were not different active pharmaceutical substances that would have to be re-approved by the national authority.

## 3. Materials and Methods

### 3.1. Materials

Tetracaine Hydrochloride (Fagron a.s., Olomouc, Czech Republic) approved by the Czech national authority State Institute for Drug Control (SÚKL) for individually prepared medicinal products (Magistral medicines), and Levofloxacin Kabi 5 mg/mL infusing solution (Fresenius Kabi, Chicago, IL, USA) approved by SÚKL, reg. number 42/117/13-C, were used as active pharmaceutical ingredients (API) for the drug release experiments. Riboflavin-5′-phosphate sodium salt dihydrate and Dextran Mw ca 500,000 were purchased from Alfa Aesar, part of Thermo Fisher Scientific, Germany, and used as delivered without any further purification. Contact lens-shaped drug carriers with diameters of 10 ± 2 mm and thicknesses of 500 µm were prepared from medical-grade atelocollagen material (the supplier’s brand is confidential and protected as a trade secret). The carriers were stored in a standard storage solution for contact lenses to prevent any damage before use. All materials were stored at a temperature of 2–8 °C.

### 3.2. UV-Vis Analysis

All prepared and investigated solutions were examined by a double-beam UV-Vis spectrometer Lambda 1050 (Perkin Elmer, Shelton, CT, USA). Absorption spectra were measured in the range of 200–600 nm using 1 mm-path cuvette or 1 cm-path cuvette, depending upon the volume of the sample. To analyze the samples quantitatively, the concentration calibration curves of both drugs were constructed for all kinds of examined sample matrices. The concentration of drug in the solutions was determined by reading the absorbance at the maximum at a typical wavelength (310 nm for Tetracaine and 287 nm for Levofloxacin), subtracting the background.

### 3.3. Drug Carrier Preparation

First, the collagen carriers were removed from the storage solution and rinsed with demineralized (DEMI) water several times, and each specimen was placed into a custom-made implant holder. The collagen carriers within the holders were placed in a standard oven (Memmert UN 55) and dried at 32 °C for 3 days (approximately 72 h). The holders avoided deformation of the shape of the carriers during the drying process. The dried collagen carriers were immersed in a fresh loading solution (25 mL) containing the required drug (2.5 wt% solutions of Tetracaine or/and Levofloxacin) for 24 h. The total extractable amount of loaded drugs in carriers was estimated using UV-Vis spectrometry after 72 h of release. On average, (2.4 ± 0.1) mg in the case of Tetracaine and (0.50 ± 0.02) mg in the case of Levofloxacin loadings were achieved. This procedure was adapted from the previous study [61]. Then, the drug carriers were crosslinked. For this purpose, a mixture containing 10 mg of Riboflavin phosphate in 10 mL of Dextran (20 wt% solutions) was prepared [37]. Crosslinking was performed with a UV lamp UVP UVLMS-38 (Analytik Jena US, Upland, CA, USA) at the wavelength of 365 nm and power ca 5 mW/cm^2^, for 15 min. Three drops of Riboflavin/Dextran solution were initially dripped on drug carriers using a plastic Pasteur pipette, and later one drop of solution was applied every 5 min. Each drop had a volume of approximately 10 µL. After 15 min, the residual Riboflavin/Dextran solution was rinsed off with DEMI water. Crosslinked collagen drug carriers were stored in the loading solution before their use in further drug release experiments.

The investigation of the prepared barrier on the surface of the collagen carrier was done with a dispersive confocal Raman microscope (Nicolet DXR Raman microscope from Thermo Scientific, Waltham, MA, USA). The excitation laser with a wavelength of 780 nm was used. Spectra were measured with exposure time 1 s and 240 scans. A crosslinked surface layer was analyzed on a sample after the crosslinking procedure; however, without the drug involved. Spectra were collected from the surface (0 µm) to the depth of 200 μm to obtain a depth profile of crosslinking.

For the drug release and release efficiency experiment, corneal tissues taken from a dead porcine eye were used. Porcine corneas were originally taken from enucleated eyeballs from the Czech breeding of a standard slaughter weight of 120 kg and age of 4 months. Cornea specimens were stored in a solution for corneal storage Eusol-C (AlchimiA, Ponte San Nicolò, Italy).

### 3.4. Model Device and Drug Release Experiment

**Figure 10:**
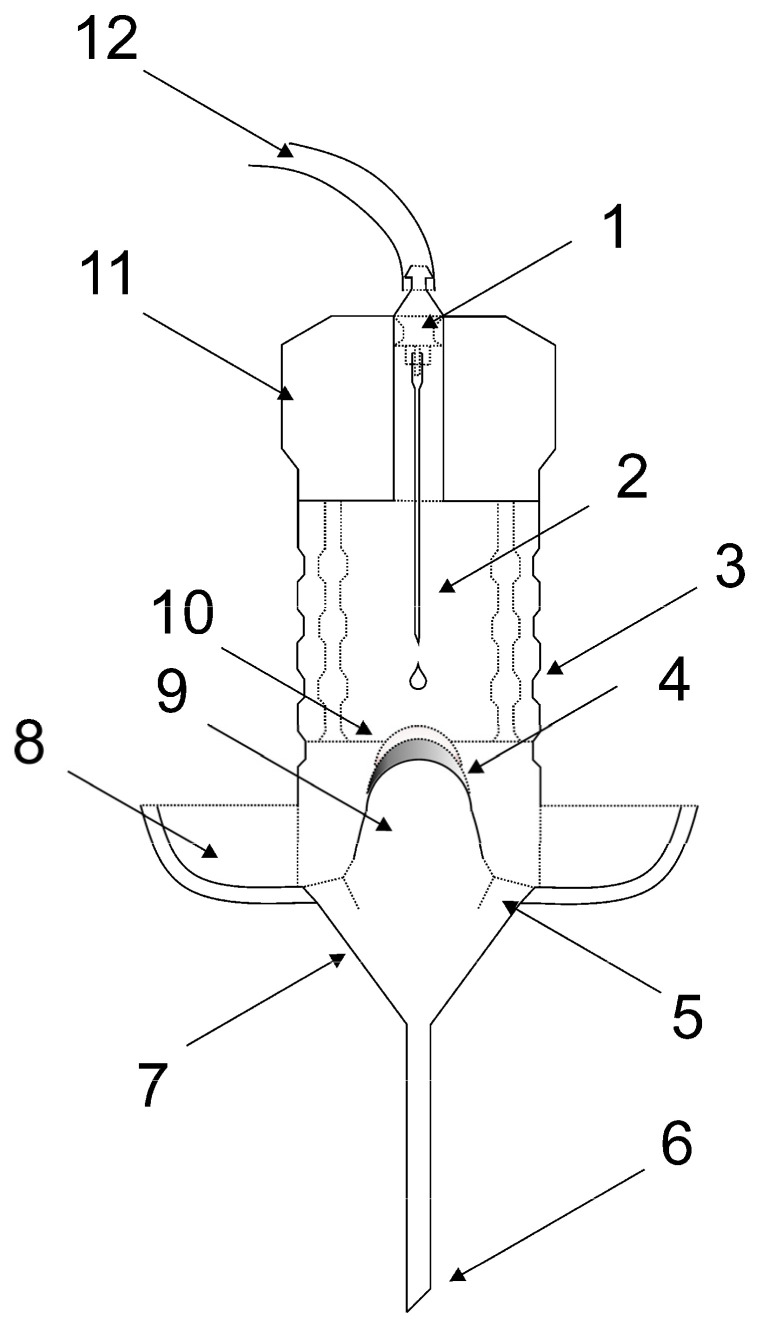
The scheme of the model device with the drug carrier and cornea specimens inserted. The model consists of (1) Luer lock adapter, (2) needle for drop generation, (3) cylindrical moisturizing chamber with perforated wall wrapped in a soaking fabric ensuring humidity, (4) cornea specimen, (5) collecting channel guiding the liquid to the funnel, (6) outlet of the funnel, (7) collecting funnel, (8) water reservoir for moisturization, (9) eyeball shaped specimen holder with a spherical cap, (10) contact lens-shaped drug carrier specimen, (11) sealing lid, and (12) inlet tubing from the syringe pump. For more details, dimensions and a three-dimensional view, see Figure S1 in Supplementary information.

We have developed a model for monitoring the drug release from the collagen carrier under simulated conditions of eyeball geometry and eye lacrimation at a physiological rate. The model was 3D printed from modified polyester (CPE HG100, Filamentum, Czech Republic). A schematic is presented in Figure 10. Dimensions and descriptions of individual parts are given in Supplementary information in Figure S1. 

The model does not include the effect of the eyelids blinking. Therefore, the moisture is ensured by the cylindrical moisturizing chamber wrapped in a water-soaked fabric to avoid drying of the sample. The model can be used in two ways. First, the collagen drug carrier can be placed on the spherical cap of the sample holder and tested. The buffer solution (PBS) simulating tears is dropped on the specimen at the physiological lacrimation rate. In the second arrangement, a cornea specimen from a dead porcine eye is placed on the spherical cap, and the collagen drug carrier is placed on top of the cornea specimen. Note that the porcine cornea must be rinsed thoroughly from the storage solution before the experiment to prevent later interference with the solution containing the released drug. Then, the tear simulation is performed as in the first case.

**Figure 11:**
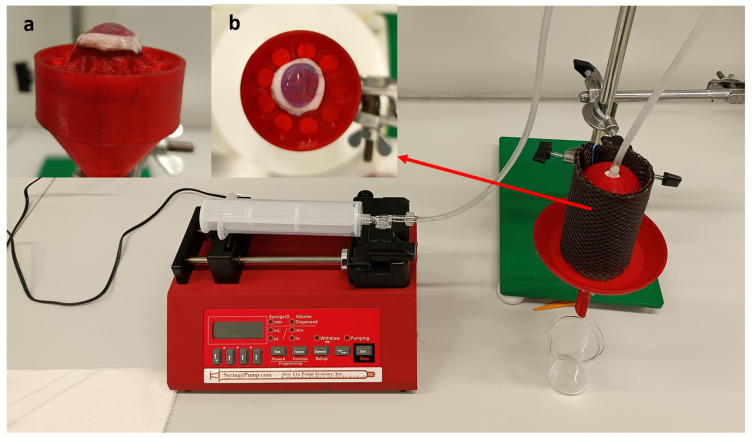
Actual view of the apparatus prepared for the drug release experiment, equipped with a syringe pump. The brown fabric wrapped around the sample chamber is soaked with water to maintain moisture around the carrier and cornea. The insets show the cornea and collagen drug carrier placed in the holder (**a**) side view and (**b**) top view.

A syringe pump (NE-1000 One Channel Programmable Syringe Pump, New Era Pump System Inc., Farmingdale, NY, USA) was used to ensure the dripping of PBS at an appropriate physiological flow rate (12 µL/min), simulating lacrimation of the eye. The volume of the used syringe was 6 mL. The PBS dripped from a needle located directly above the surface of the collagen drug carrier sample. The liquid flowing from the surface of the sample was collected in a container under the holder. After eight hours of dripping, the porcine cornea was placed in a phosphate buffer solution (20 mL), where it was leached for 24 h to analyze the total amount of adsorbed API. The actual view of the apparatus is shown in Figure 11, with details of the cornea placement. The soaked fabric (brown color in Figure 11) ensured the humidity. The temperature during the release experiments was 25 °C. Accepting a compromise in testing temperature was necessary due to the danger of dead corneal tissue decay, the physiological temperature of the anterior surface of human cornea, the average temperature in devices such as contact lenses when applied, and the practical demands of common laboratory work.

Both the fluid collected during the experiment and the corneal leachate were analyzed with UV-Vis spectrometry using the 1 mm-path cuvette. Obtained concentration values were recalculated to reflect the released amounts of API in question. The experiments were done in triplicate. Calibration curves of both API and representative absorption spectra are in Supplementary Information in Figures S2–S5.

### 3.5. HPLC Analysis of API

High-performance liquid chromatography with mass detection (HPLC-QTOFMS) was performed on a 1260 Infinity LC system (Agilent Technologies, Santa Clara, CA, USA). Chromatographic separation of the components of the samples was carried out on a ZORBAX Extend C18 column (50 mm × 2.1 mm, 1.8 µm) (Agilent Technologies, Santa Clara, CA, USA) at a flow rate of 0.300 mL·min^−1^ and temperature 35 °C. The mobile phase consisted of 0.1% formic acid in water (A) and acetonitrile (B) with isocratic elution (79:21; A:B). The total running time was 7 min for each sample, and the sample injection volume equaled 5 µL. 

Detection was performed on a quadrupole time of flight mass spectrometer (6530 Q-TOF, Agilent Technologies, Santa Clara, CA, USA) employing an electrospray ion (ESI) source set to the positive mode. Samples were measured as [M^+^H]^+^ molecule ions. The mass spectrometer operated under the following parameters: capillary voltage 4500 V, nebulizer pressure 40 psig, drying gas 8 L·min^−1^, and gas temperature 300 °C. Mass spectra were acquired over the *m*/*z* 100–1500 range at a scan rate of 2 scan·s^−1^. Accurate mass measurements were obtained via a calibrating solution involving the use of internal reference masses (TFA anion [C_2_O_2_F_3_(NH_4_)] at *m*/*z* 112.985587, and HP-0921 [hexakis-(1H,1H,3H-tetrafluoropentoxy)-phosphazene] (C18H18O6N3P3F24) at *m*/*z* 1033.988109). Data were recorded and processed in MassHunter software v.B.05.01 (Agilent Technologies).

## 4. Conclusions

The work presents a new solution for a local anesthetic (Tetracaine) and antibiotic (Levofloxacin) delivery suitable for treating conditions after eye surgery. The drug carrier was prepared in the form of contact lens-shaped collagen material that gradually releases the drug effectively and efficiently into the corneal tissue, according to the prolonged release profile of pseudo-first-order kinetics. Compared with antibiotic and pain-relieving eyedrops, this new drug delivery form is considered many times more effective and could work up to 72 h. It would be necessary to use an excessive volume of the eyedrops (approx. 30 drops per hour) to achieve the same dose as delivered gradually by our collagen drug carrier. 

As a prerequisite, an original 3D-printed device was designed and prepared, and a method for controlled drug release testing that simulates conditions close to the geometry and physiological lacrimation of the human eye was developed. Hence, drug carriers can be effectively tested with or without a dead porcine cornea as a recipient of the drugs, which significantly eliminates the need for live animals for testing in vivo.

## Data Availability

Data is contained within the article and Supplementary Material.

## Supplementary Materials

The following supporting information can be downloaded at: https://www.mdpi.com/article/10.3390/ph16040505/s1. Figure S1 The cornea holder prepared using 3D printer—(a) its individual parts with dimensions, (b) individual parts assembled together; Figure S2 Calibration curve of Tetracaine, measured in the maximum of absorption peak at 310 nm; Figure S3 Calibration curve of Levofloxacin, measured in the maximum of absorption peak at 287 nm; Figure S4 Representative Tetracaine absorption spectra achieved during the release experiment; Figure S5 Representative Levofloxacin absorption spectra achieved during the release experiment; Table S1 Detection limits; Figure S6 Baseline shown in areas of interest for drugs detection with marked absorption maxima for both API; Figure S7 Tetracaine release profiles from unmodified (black) and surface-crosslinked (red) collagen drug carriers with kinetic model and its parameters; Figure S8 Levofloxacin release profiles from unmodified (black) and surface-crosslinked (red) collagen drug carriers with kinetic model and its parameters; Figure S9 Tetracaine (MS scan); Figure S10 Tetracaine (MS scan, extracted ion 265.1959 *m*/*z*); Figure S11 Tetracaine (MS scan, extracted ion 265.1959 *m*/*z*, asterisk mean saturated detector); Figure S12 Tetracaine (MSMS scan, product ions from *m*/*z* 265.1959, CID 20.0 V) after release experiment; Figure S13 Levofloxacin (MS scan); Figure S14 Levofloxacin (MS scan, extracted ion 362.1515 *m*/*z*); Figure S15 Levofloxacin (MS scan, extracted ion 362.1515 *m*/*z*, asterisk mean saturated detector); Figure S16 Levofloxacin (MSMS scan, product ions from *m*/*z* 362.1516, CID 20.0 V) after release experiment; Figure S17 Tetracaine release profiles of drug carriers with the kinetic model and its parameters—first-order exponential kinetic model and comparison with the same exponential model including the Korsmeyer–Peppas (KP) component; Figure S18 Levofloxacin release profiles of drug carriers with the kinetic model and its parameters—first-order exponential kinetic model and comparison with the same exponential model including the Korsmeyer–Peppas (KP) component.
