# Correction: Urbánek et al. Controlled Drug Delivery Device for Cornea Treatment and Novel Method for Its Testing. *Pharmaceuticals* 2023, *16*, 505

**DOI:** 10.3390/ph18050720

**Published:** 2025-05-14

**Authors:** Pavel Urbánek, Pavol Šuly, Jakub Ševčík, Barbora Hanulíková, Ivo Kuřitka, Tomáš Šopík, Mehrdad Rafat, Pavel Stodůlka

**Affiliations:** 1 Centre of Polymer Systems, Tomas Bata University in Zlín, Trida Tomase Bati 5678, 76001 Zlin, Czech Republic; suly@utb.cz (P.Š.); j4sevcik@utb.cz (J.Š.); hanulikova@utb.cz (B.H.); kuritka@utb.cz (I.K.); sopik@utb.cz (T.Š.); 2 LinkoCare Life Sciences AB, 16975 Stockholm, Sweden; mrafat@linkocare.com; 3 NaturaLens AB, 58330 Linköping, Sweden; 4 Gemini Eye Clinic, U Gemini 360, 76001 Zlin, Czech Republic; stodulka@lasik.cz

## Text Correction

As an addition to the original publication [1], we conducted a supplementary data analysis to clearly reflect the contributions of all team members.

A correction has been made to 2. Results and Discussion, 2.2. Drug Release from the Collagen Carrier, Paragraph 4:

On the other hand, the exponential model with the Korsmeyer–Peppas (KP) component *b*·*t*^n^_r_ [57], where *b* represents the kinetic constant characteristic of the drug/polymer system, *t*_r_ is the release time, and n is the diffusional release exponent, fits the data obtained for Levofloxacin with greater accuracy (R^2^ = 0.99551)—see Figures S17 and S18. The Korsmeyer–Peppas model combined with the first-order exponential model seems to be an appropriate model for more structurally complicated substances and polymer carriers.

## Addition of Supplementary Materials

As an addition to the original publication [1], we conducted a supplementary data analysis to clearly reflect the contributions of all team members and updated the Supplementary Materials. The newly added Supplementary Materials part appears below:

Figure S17 Tetracaine release profiles of drug carriers with the kinetic model and its parameters—first-order exponential kinetic model and comparison with the same exponential model including the Korsmeyer–Peppas (KP) component; Figure S18 Levofloxacin release profiles of drug carriers with the kinetic model and its parameters—first-order exponential kinetic model and comparison with the same exponential model including the Korsmeyer–Peppas (KP) component.

## Addition of an Author

In the original publication [1], Mehrdad Rafat was not included as an author. Mehrdad Rafat belongs to the new affiliations 2 and 3:

2 LinkoCare Life Sciences AB, 16975 Stockholm, Sweden; mrafat@linkocare.com
3 NaturaLens AB, 58330 Linköping, Sweden

The corrected Author Contributions statement appears here.

Conceptualization: P.U. and I.K.; investigation: P.U., B.H., J.Š., P.Š., T.Š., P.S., M.R. and I.K.; methodology: P.U., P.S. and I.K.; formal analysis P.U., B.H. and I.K.; visualization: P.U., B.H., J.Š. and I.K.; resources: M.R.; data analysis: P.U. and M.R.; writing—original draft: P.U., B.H., P.Š. and I.K.; writing—review and editing: P.U. and I.K.; funding acquisition: P.U., I.K. and P.S. All authors have read and agreed to the published version of the manuscript.

## Conflicts of Interest

In the original publication [1], the Conflicts of Interest statement of Mehrdad Rafat was not included. The updated Conflicts of Interest should read as follows: Author Mehrdad Rafat was employed by the companies LinkoCare Life Sciences AB and NaturaLens AB. The remaining authors declare that the research was conducted in the absence of any commercial or financial relationships that could be construed as potential conflicts of interest.

The authors state that the scientific conclusions are unaffected. This correction was approved by the Academic Editor. The original publication has also been updated.
