# Supplementary material for: Controlled Drug Delivery Device for Cornea Treatment and Novel Method for Its Testing
Source: Pharmaceuticals (Basel). 2023 Mar 28;16(4):505. doi: 10.3390/ph16040505 (PMC10143253; doi:10.3390/ph16040505)
Supplement: Supplementary file 1 [file pharmaceuticals-16-00505-s001.zip › pharmaceuticals-2166719-supplementary.pdf]

Supplementary information

Controlled drug delivery device for cornea treatment and novel method for its testing

[1*]Pavel Urbánek, [1]Pavol Šuly, [1]Jakub Ševčík, [1]Barbora Hanulíková, [1]Ivo Kuřitka, [1]Tomáš Šopík, [2,3]Mehrdad Rafat, and [4]Pavel Stodůlka

[1]Centre of Polymer Systems, Tomas Bata University in Zlín, trida Tomase Bati 5678, Zlin 76001, Czech Republic

[2]LinkoCare Life Sciences AB, 16975 Stockholm, Sweden

[3]NaturaLens AB, 58330 Linköping, Sweden

[4]Gemini Eye Clinic, U Gemini 360, 760 01 Zlin – Priluky, Czech Republic

*Corresponding author: urbanek@utb.cz

Detailed dimensions of constructed holder

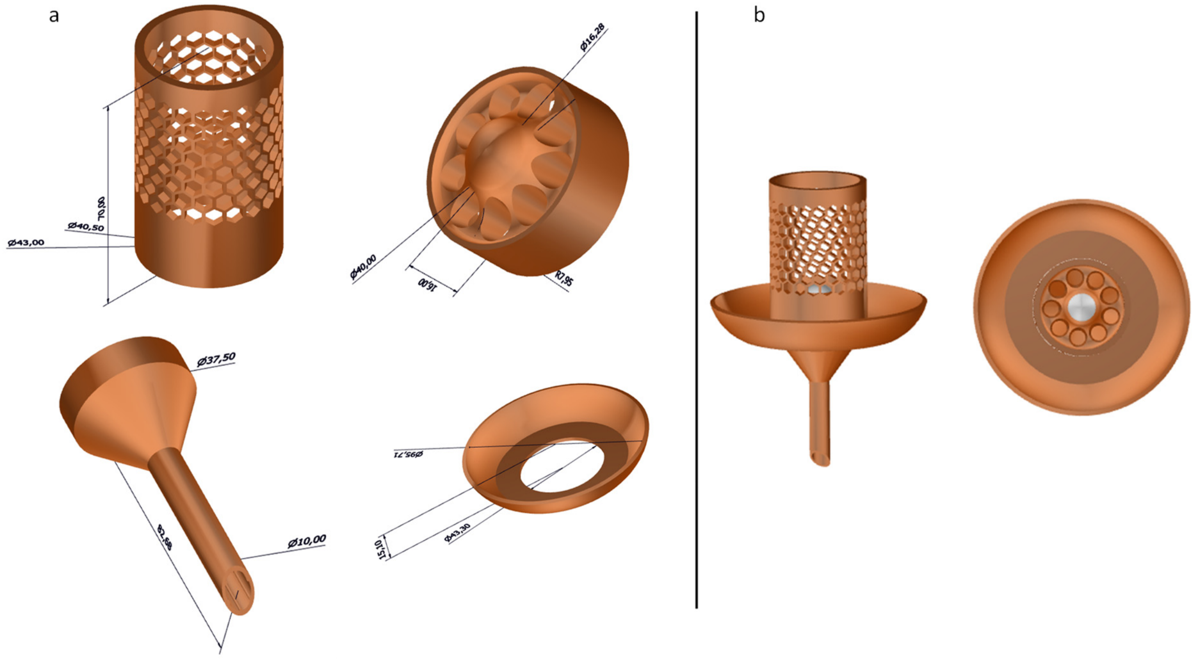

Figure S1 The cornea holder prepared using 3D printer – a) its individual parts with dimensions, b) individual parts assembled together.

UV-Vis characterization

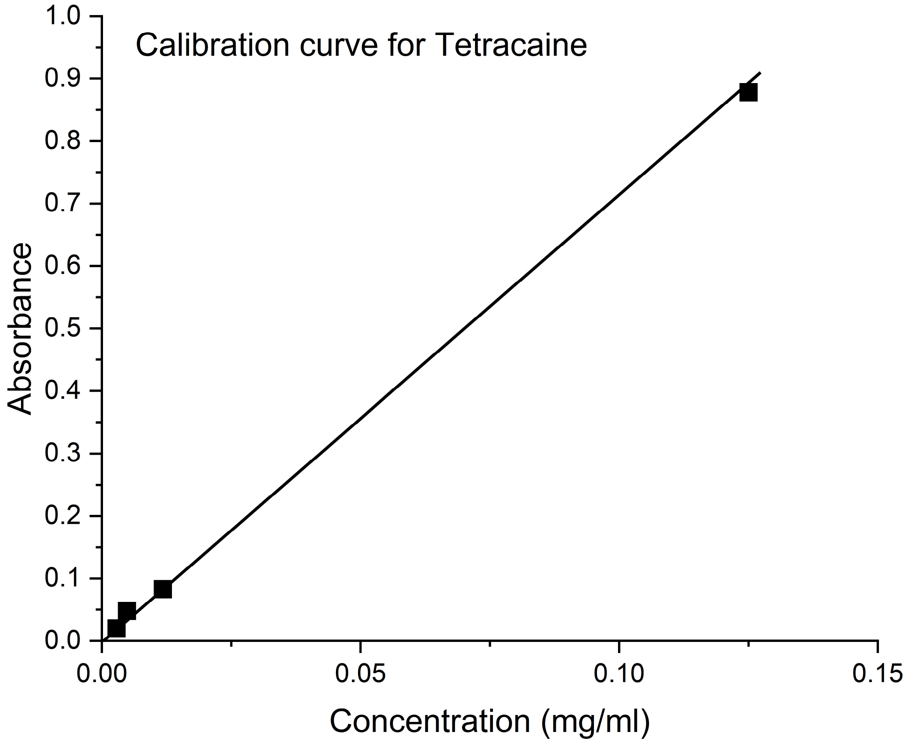

Figure S2 Calibration curve of Tetracaine, measured in the maximum of absorption peak at 310 nm.

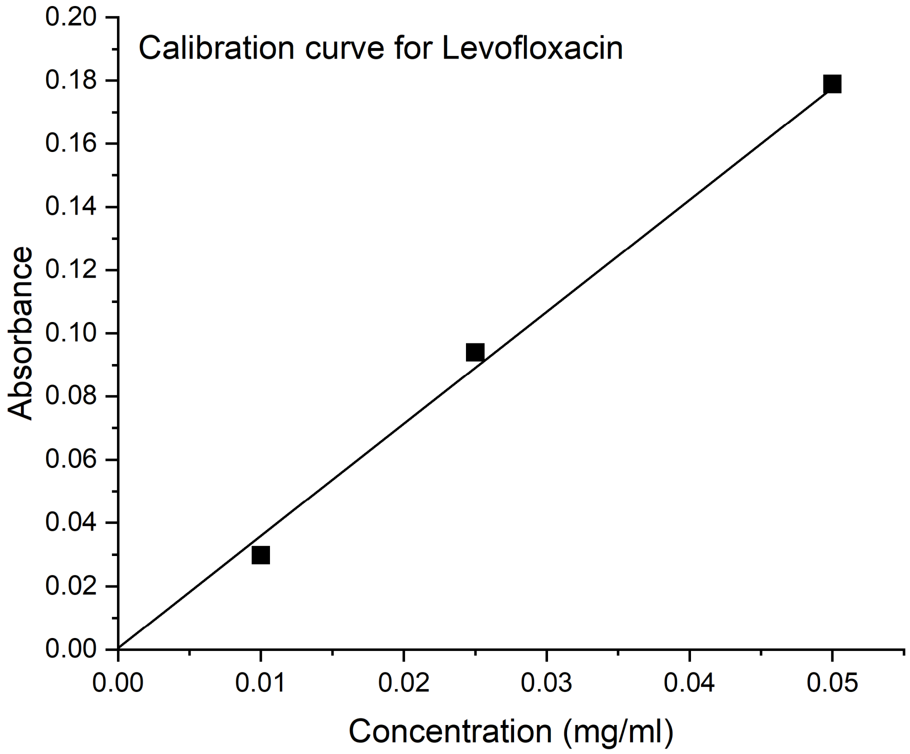

Figure S3 Calibration curve of Levofloxacin, measured in the maximum of absorption peak at 287 nm.

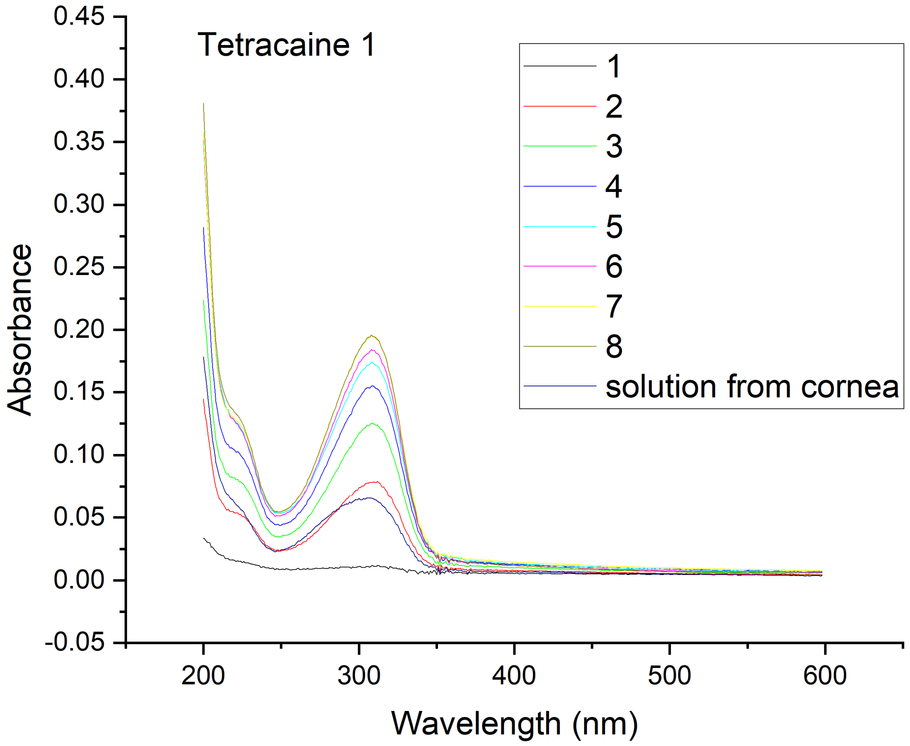

Figure S4 Representative Tetracaine absorption spectra achieved during the release experiment.

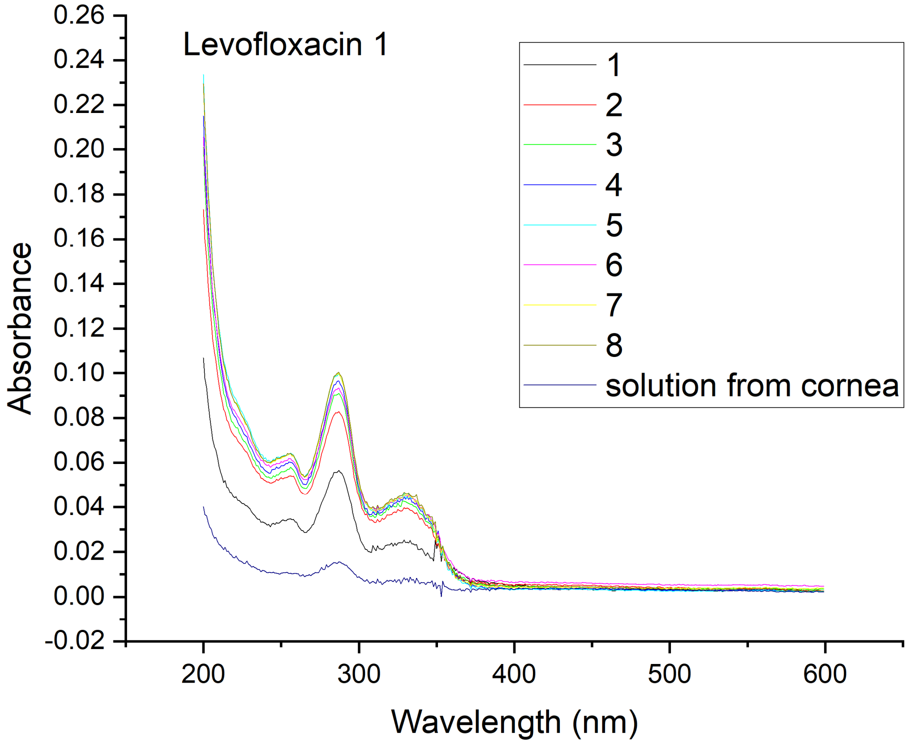

Figure S5 Representative Levofloxacin absorption spectra achieved during the release experiment.

Detection limit of UV-Vis spectrometry

Peak-to-peak value represents a value of signal noise in which the lowest noise peak is subtracted from the highest noise peak. This subtraction indicates the maximum amount of noise that could interfere the signal of the analyte in the spectrum. For the region between 307 and 312 nm, where Tetracaine has an absorption band, the peak-to-peak value is 0.00178201, for the region 284–291 nm, where Levofloxacin

has the absorption maximum, the peak-to-peak value is 0.00064517. From the measurements for the calibration curves, it follows that the absorbance of the solution with a concentration of 1 µg/ml is about 0.007 for Tetracaine and 0.0036 for Levofloxacin. It can therefore be stated that the detection limit of the method is at least 1 µg/ml.

Table S1 Detection limits.

Region [nm]	Peak-to-Peak		Absorption of solution with concentration 1 µg/ml
307–312	0.00178201	Tetracaine	0.007026
284–291	0.00064517	Levofloxacin	0.003578

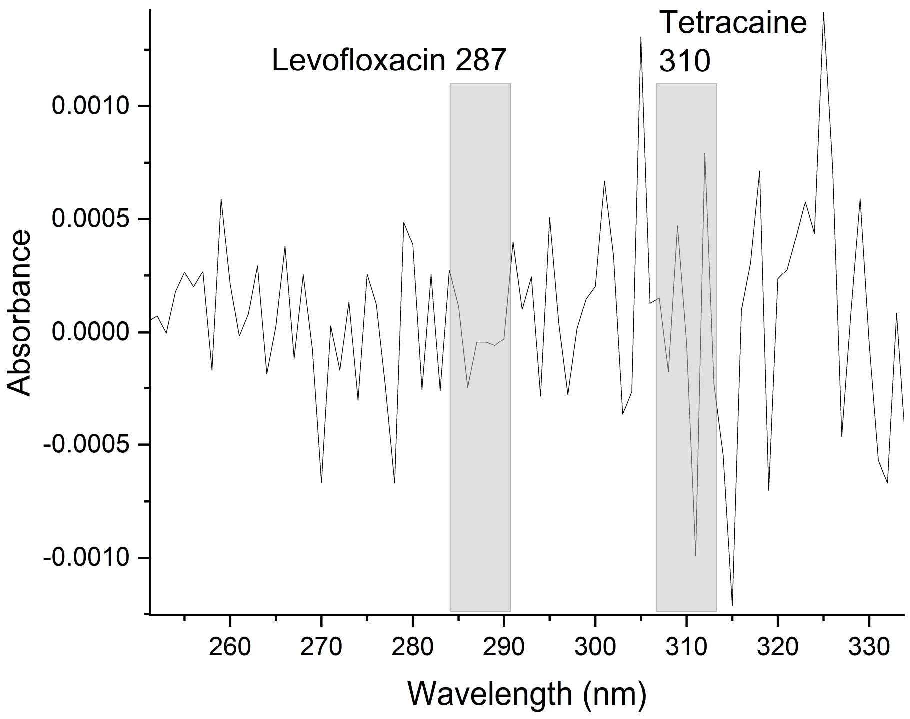

Figure S6 Baseline shown in areas of interest for drugs detection with marked absorption maxima for both API.

Kinetic models of released API

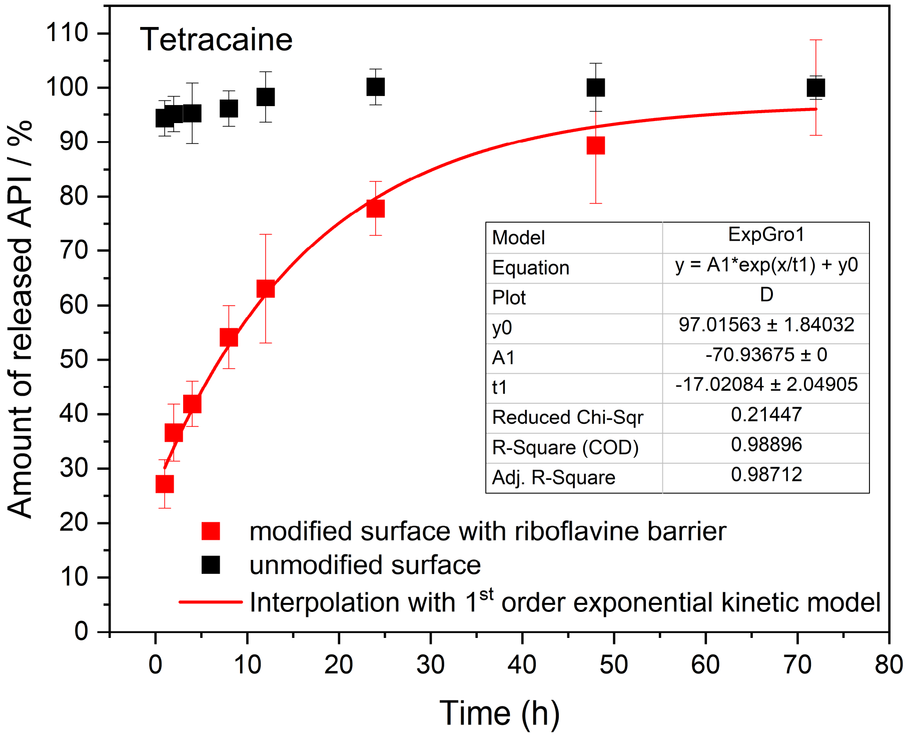

Figure S7 Tetracaine release profiles from unmodified (black) and surface-crosslinked (red) collagen drug carriers with kinetic model and its parameters.

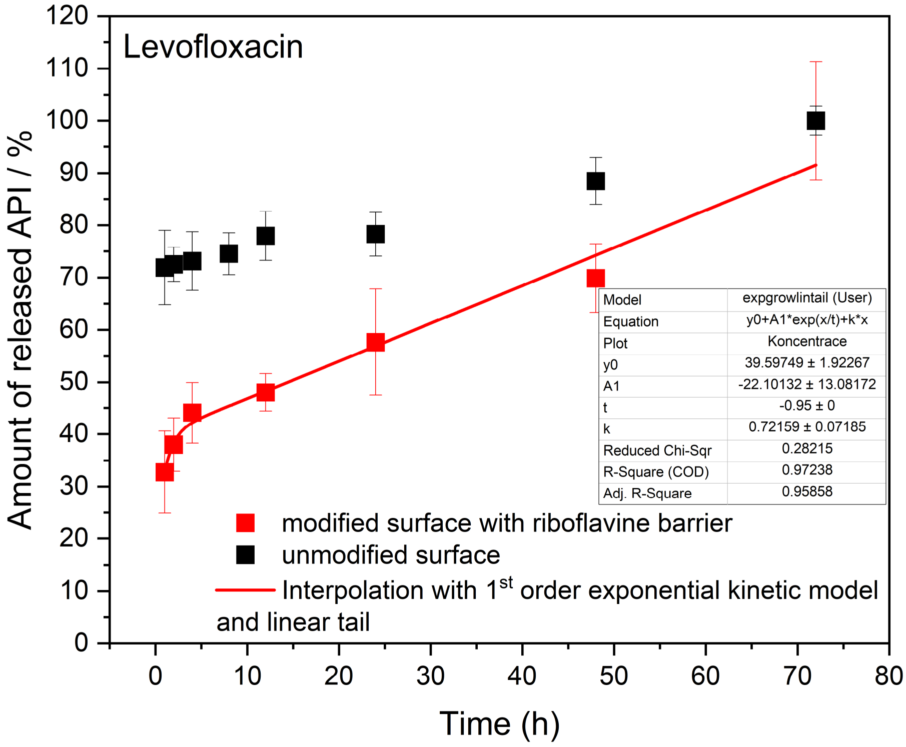

Figure S8 Levofloxacin release profiles from unmodified (black) and surface-crosslinked (red) collagen drug carriers with kinetic model and its parameters.

HPLC analysis of API and API released from carrier

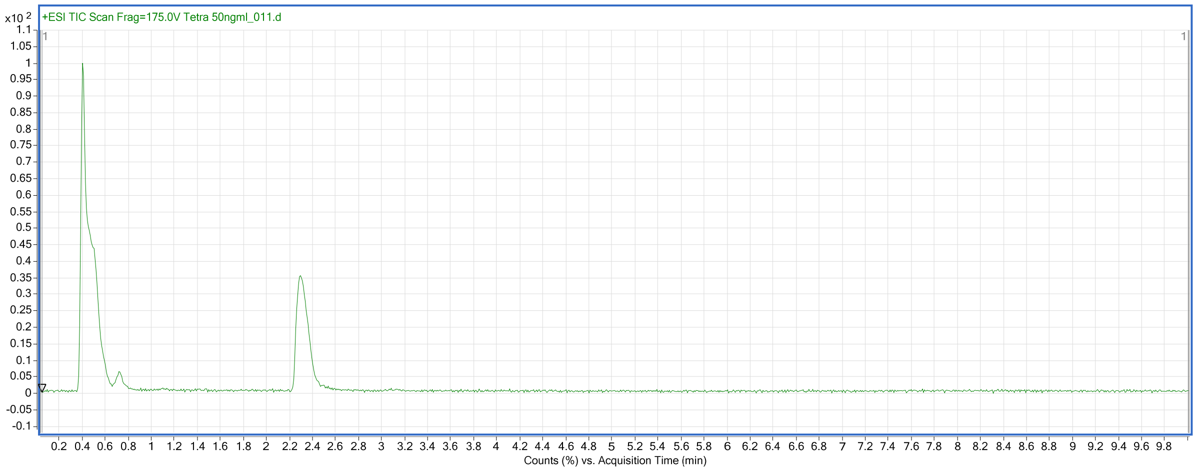

Figure S9 Tetracaine *(MS scan).*

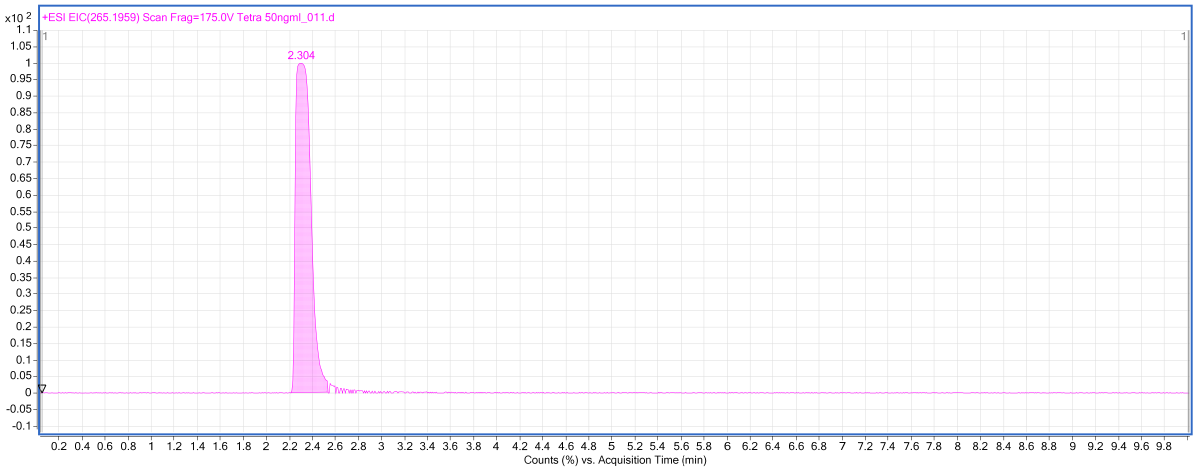

Figure S10 Tetracaine *(MS scan, extracted ion 265.1959 m/z).*

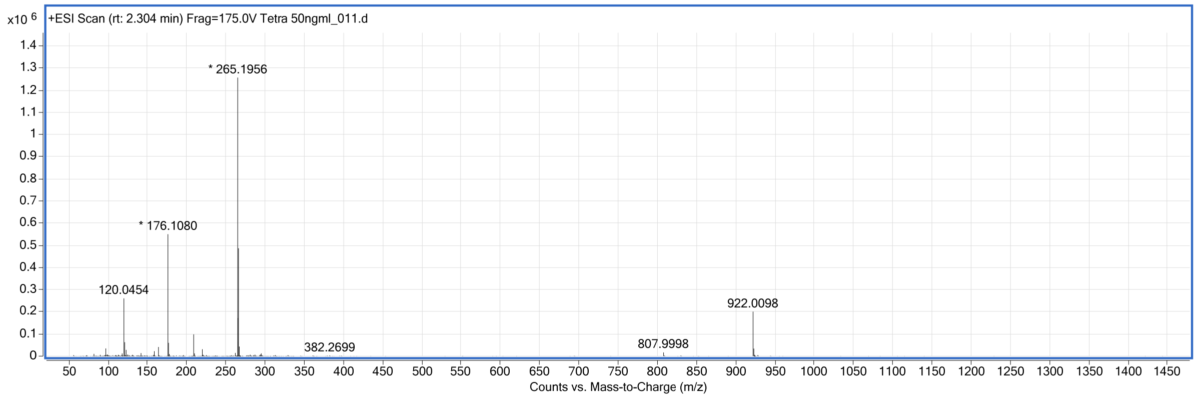

Figure S11 Tetracaine *(MS scan, extracted ion 265.1959 m/z, asterisk mean saturated detector).*

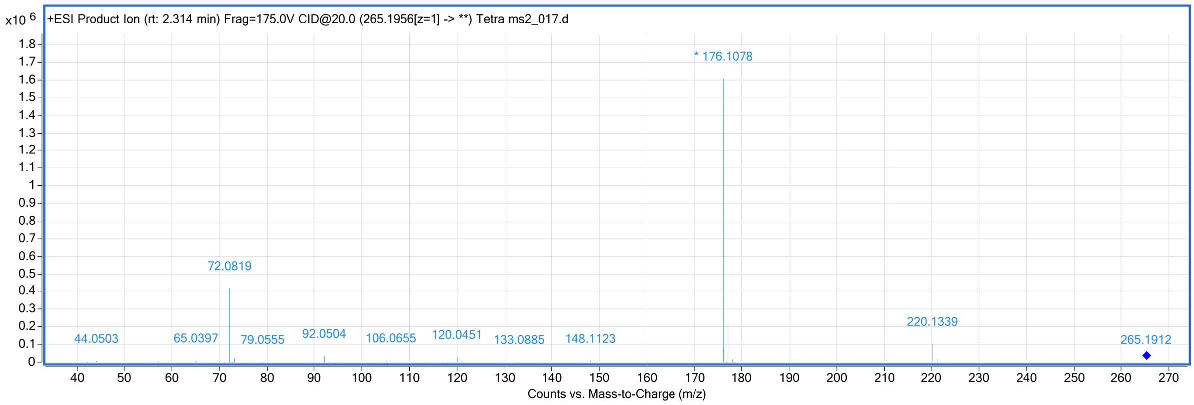

Figure S12 Tetracaine *(MSMS scan, product ions from m/z 265.1959, CID 20.0 V) after release experiment.*

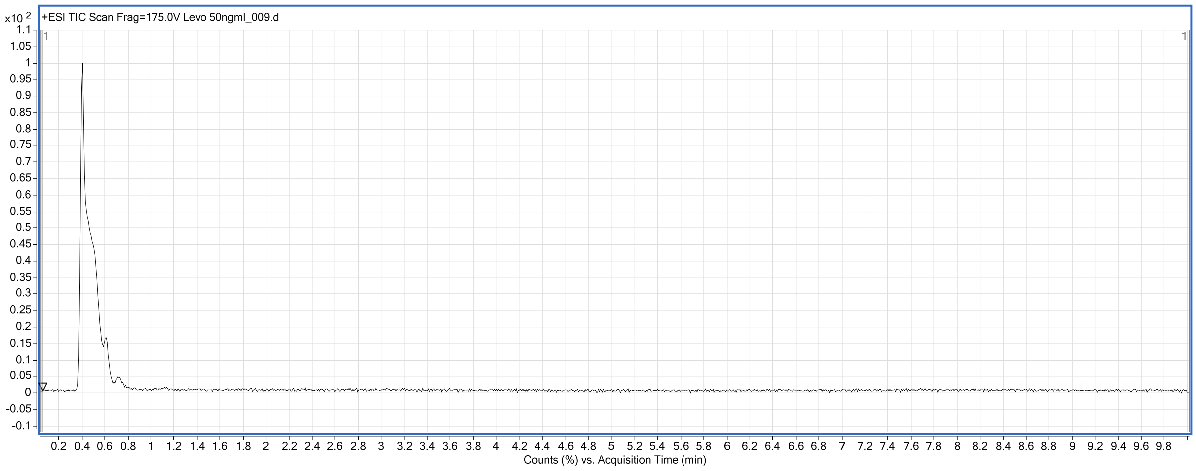

Figure S13 Levofloxacin (MS scan).

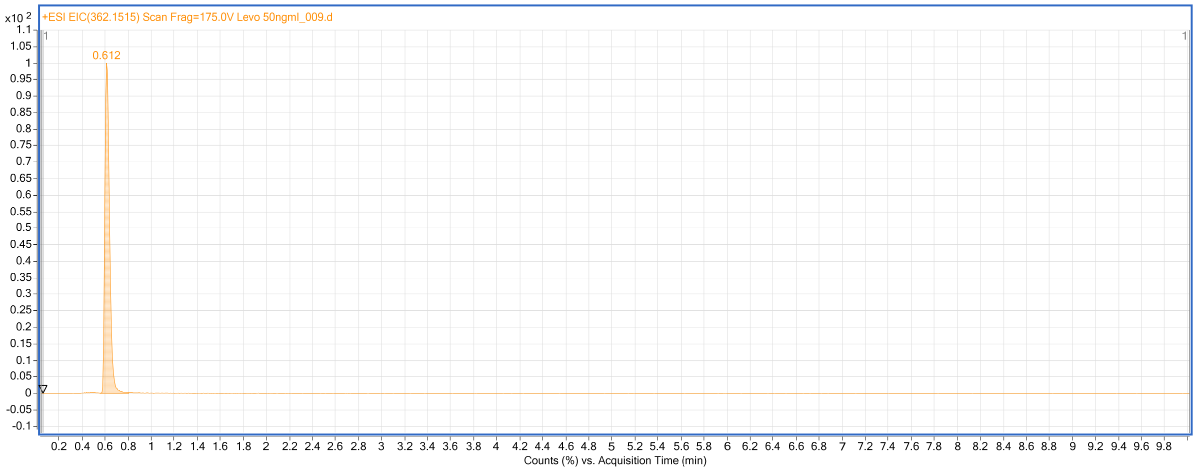

Figure S14 Levofloxacin *(MS scan, extracted ion 362.1515 m/z).*

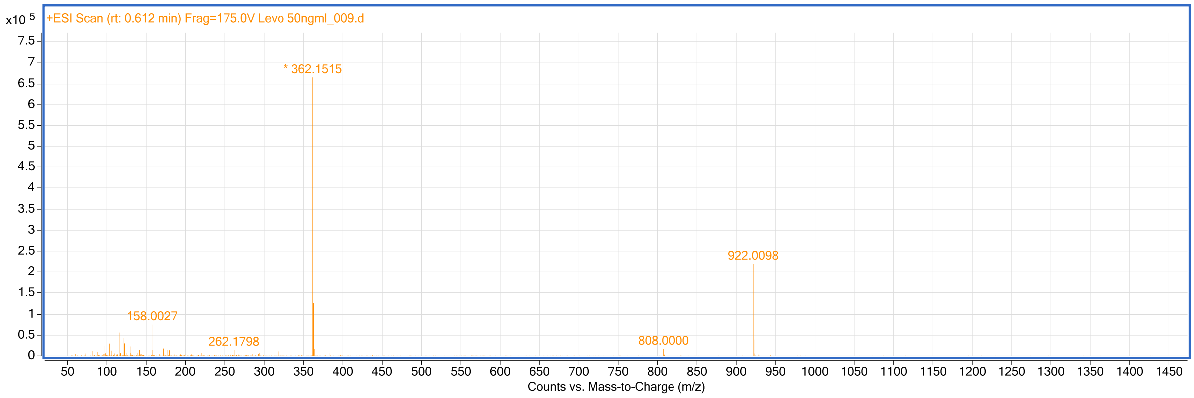

Figure S15 Levofloxacin (MS scan, extracted ion 362.1515 m/z, asterisk mean saturated detector).

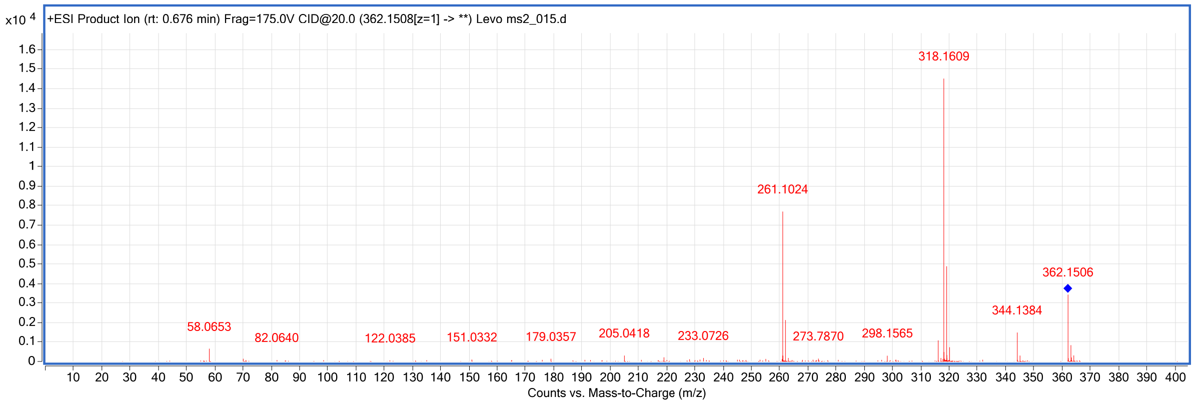

Figure S16 Levofloxacin (MSMS scan, product ions from m/z 362.1516, CID 20.0 V) after release experiment.

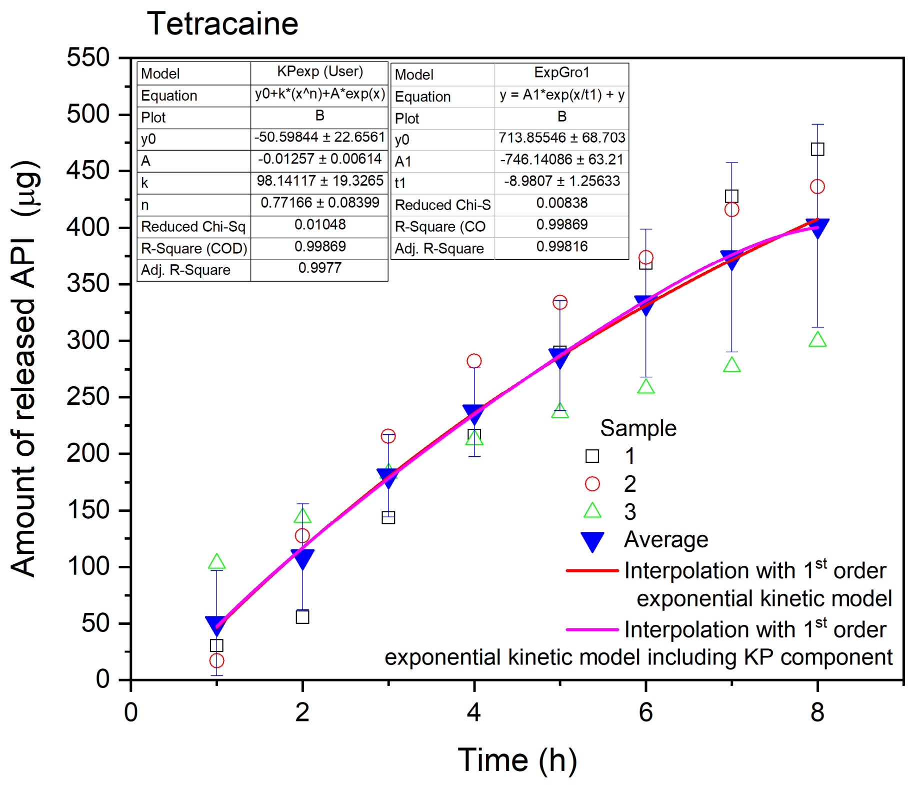

Figure S17 Tetracaine release profiles of drug carriers with the kinetic model and its parame-ters—first-order exponential kinetic model and comparison with the same exponential model including the Korsmeyer–Peppas (KP) component.

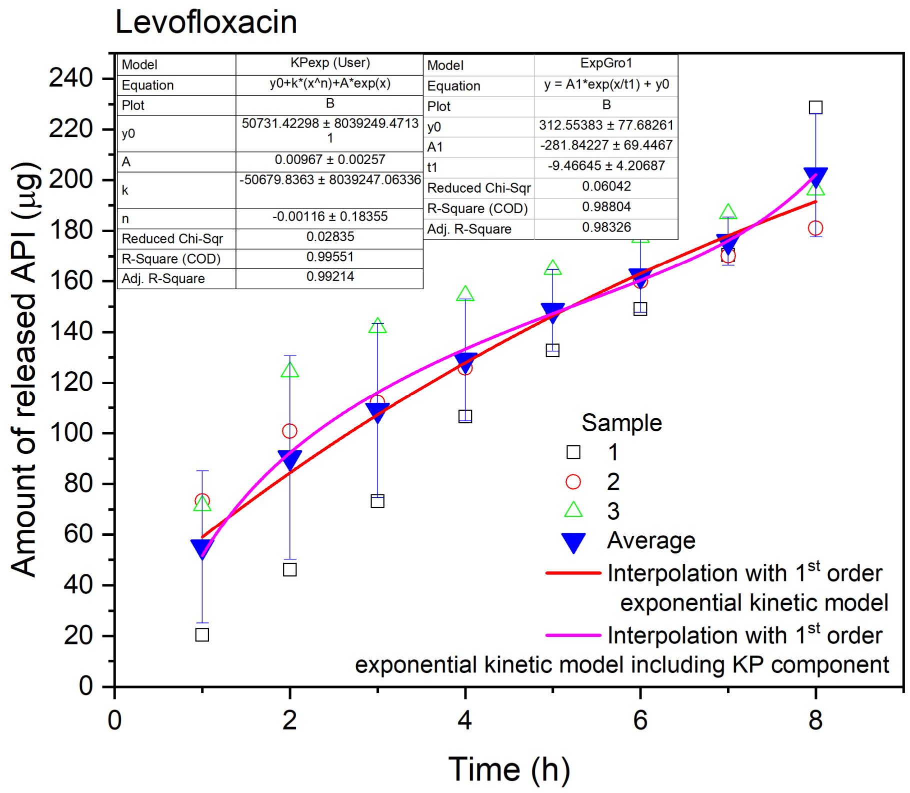

Figure S18 Levofloxacin release profiles of drug carriers with the kinetic model and its parame-ters—first-order exponential kinetic model and comparison with the same exponential model including the Korsmeyer–Peppas (KP) component.
